# A Rare Case of Intrapulmonary Sequestration With Multiple Feeding Arteries and an Anomalous Pulmonary Vein Draining Into the Azygos Vein

**DOI:** 10.7759/cureus.15628

**Published:** 2021-06-13

**Authors:** Ramakanth Pata, Nway Nway, Tsering Dolkar, Htun M Aung, Meet Patel

**Affiliations:** 1 Pulmonary Medicine, Interfaith Medical Center, New York, USA; 2 Internal Medicine, Interfaith Medical Center, New York, USA

**Keywords:** intralobar sequestration, intrapulmonary sequestration, extrapulmonary sequestration, extralobar sequestration, feeding arteries, tracheobronchial tree, ils, els

## Abstract

Pulmonary sequestration is an isolated mass of lung tissue that has no identifiable bronchial communication and that receives its blood supply from one or more anomalous systemic arteries. The feeding vessel is the aorta or its major vessels and venous drainage usually is to the pulmonary veins to the left atrium. We present a rare case of intralobar sequestration in a 65-year-old man with multiple feeding arteries from the aorta and partial anomalous venous return draining into the azygos vein. He remained asymptomatic and this anomaly was detected incidentally when computed tomography (CT) scan of the chest with contrast was done to rule out pulmonary embolism.

## Introduction

Pulmonary sequestration is a rare lung finding with an incidence of 0.1% [1]. It refers to a congenital malformation of a lung segment that lacks communication with the tracheobronchial tree and derives blood supply from systemic circulation rather than pulmonary circulation. Because of lack of communication to the airway, it is often non-functional. It is thought to be secondary to an anomaly during foregut embryogenesis. Two types of sequestrations have been described, based on the encasement by pleura and feeding artery with its draining vein. These include intralobar (ILS) or intrapulmonary and extralobar (ELS) or extrapulmonary [2]. ELS is less common (15-25%), diagnosed in newborns presenting as respiratory distress, cyanosis, or cough, and usually located outside of the normal lung with its own visceral pleura. ILS is more common (75-85%), presents later in childhood or adolescence with recurrent signs of infection, or may remain asymptomatic and detected incidentally. ILS is located within the lung, most commonly in the left lower lobe, and therefore is not encased by its own pleura [2].

## Case presentation

**Figure 1:**
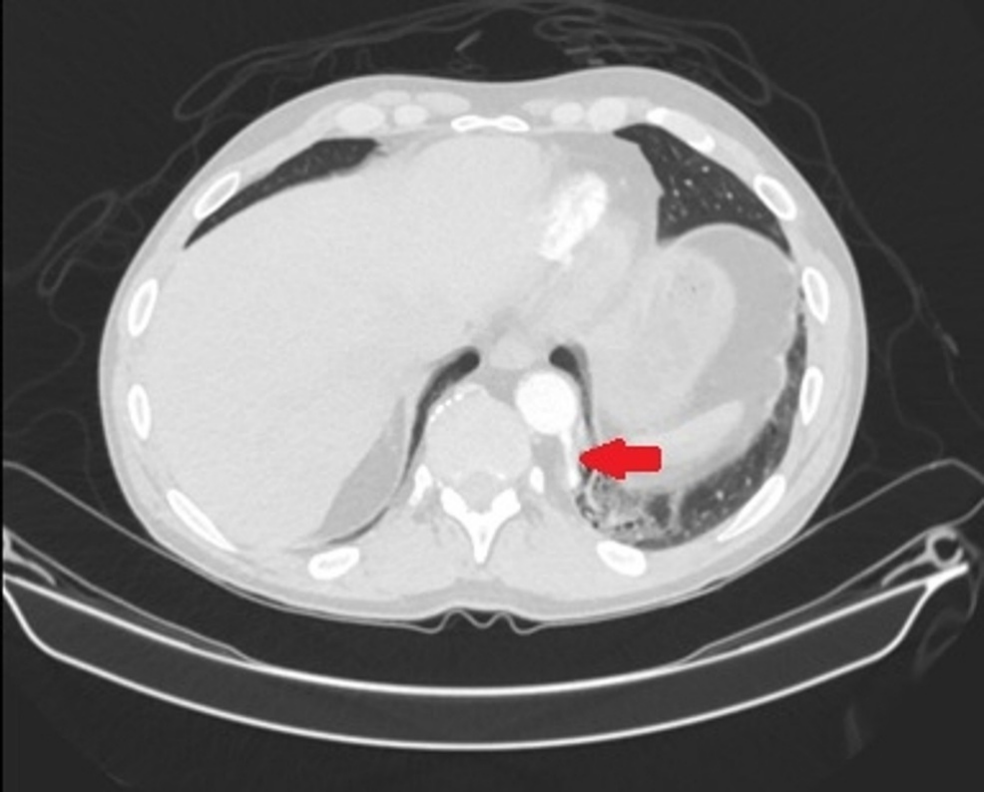
Axial CT scan of the abdomen with contrast demonstrating feeding artery arising from the aorta into intralobar sequestration. There is a subtle appreciation of more than one feeding vessel.

**Figure 2:**
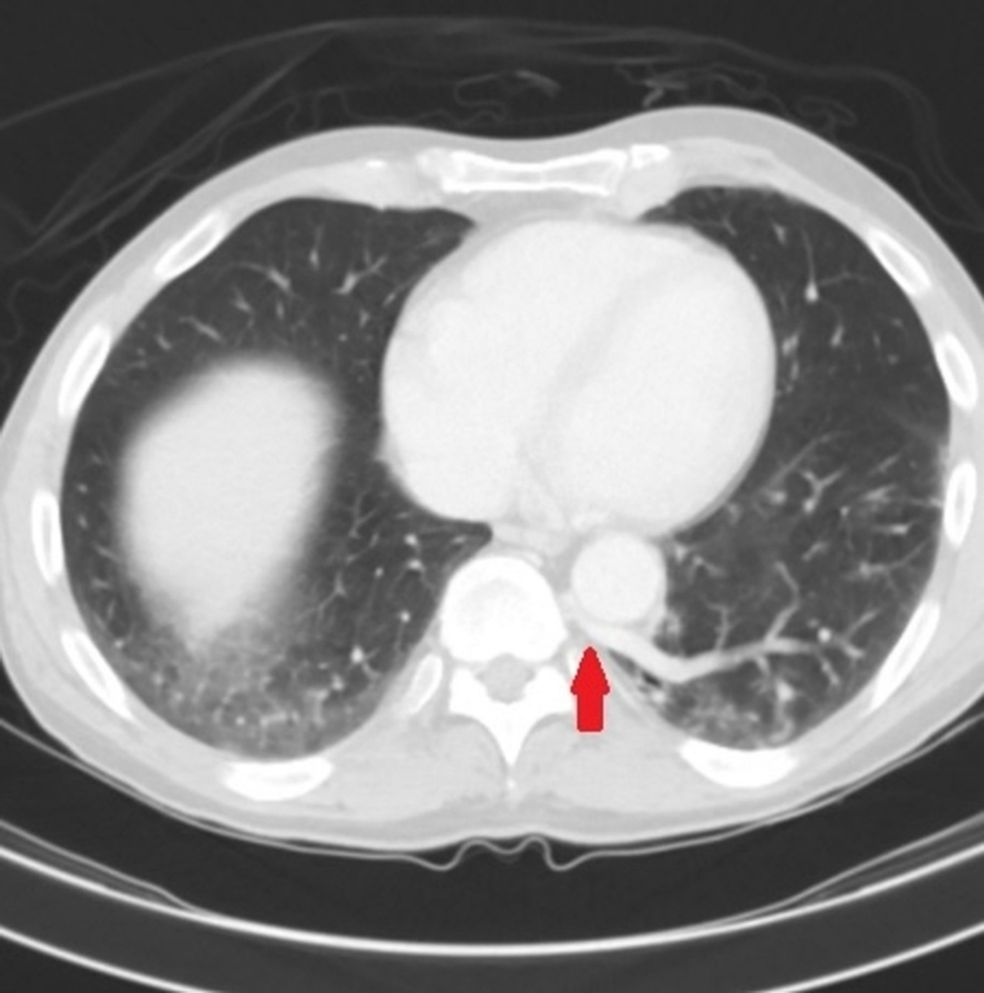
Axial CT scan of the chest with contrast demonstrating anomalous pulmonary vein arising from intralobar sequestration

**Figure 3:**
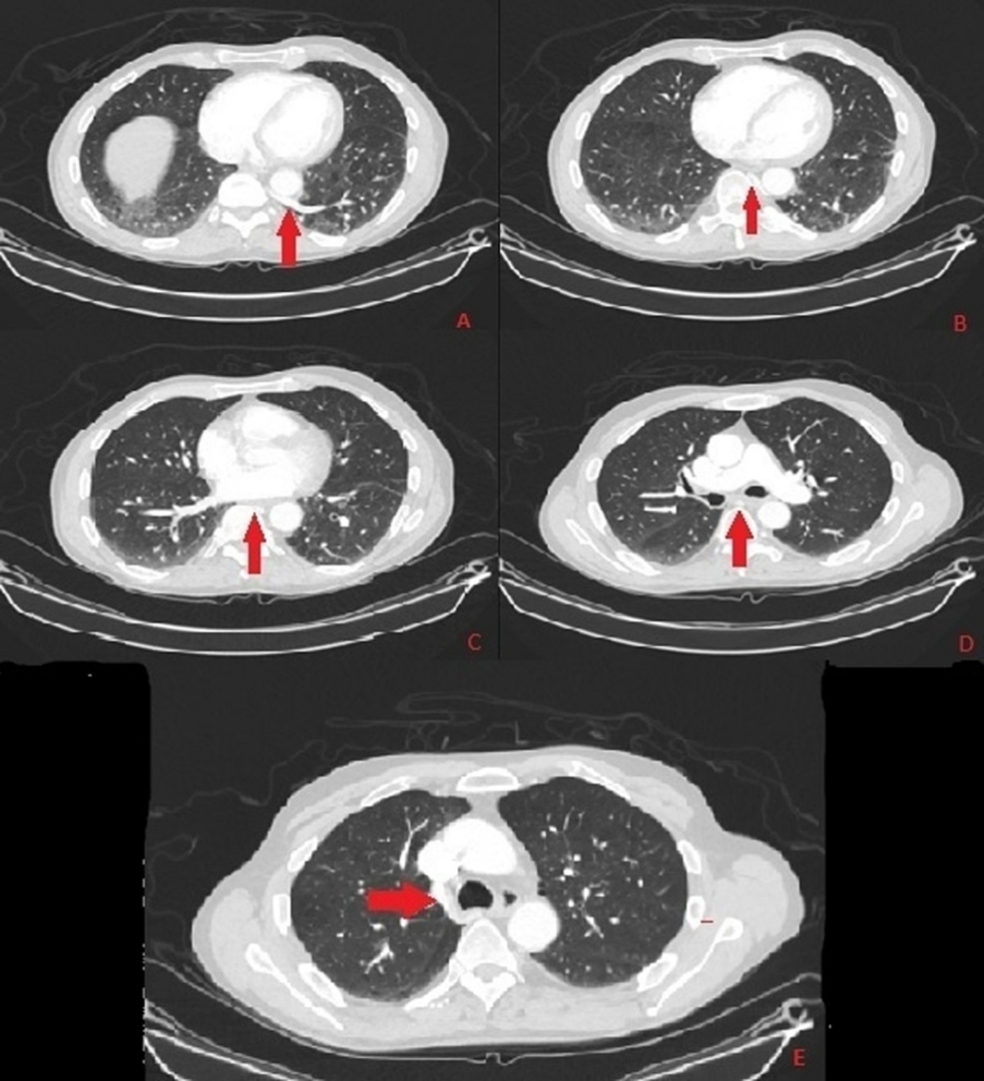
Coronal CT scan of the chest with contrast in lung window with red arrow demonstrating the course of anomalous pulmonary vein arising from intralobar sequestration (Image A) coursing on the side of the aorta (Images B, C, and D) ultimately joining azygos vein (Image E)

**Figure 4:**
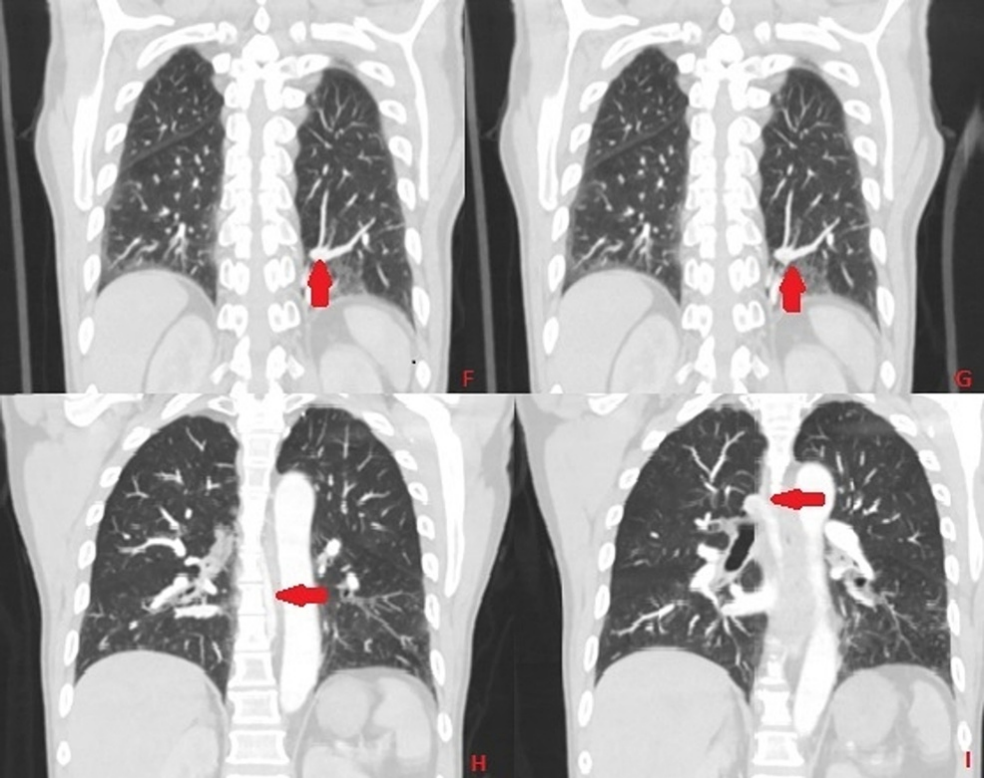
CT of the chest with contrast in the frontal plane and lung window with red arrow demonstrating the course of anomalous pulmonary vein arising from intralobar sequestration (Image F and G) coursing on the side of the aorta (Image H) ultimately joining the azygos vein (Image I)

**Figure 5:**
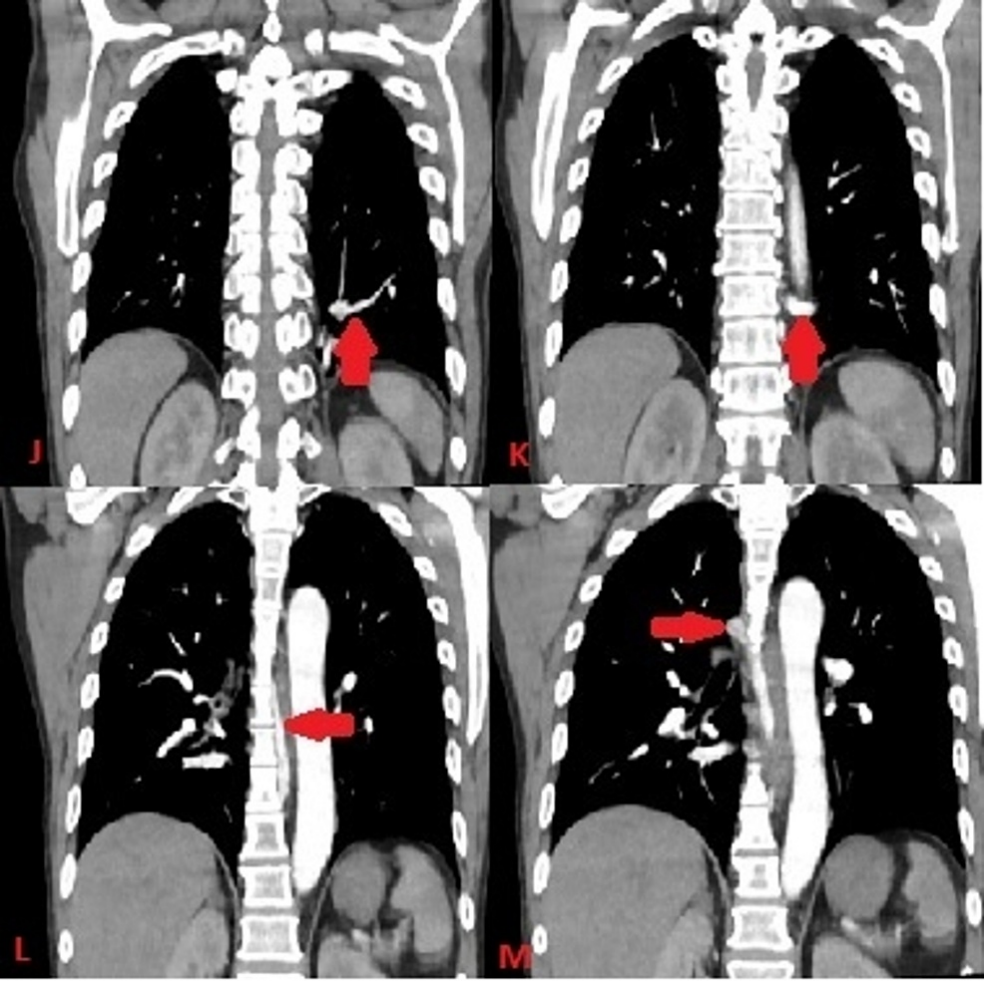
CT of the chest with contrast in the frontal plane and mediastinal window with red arrow demonstrating the course of anomalous pulmonary vein arising from intralobar sequestration (Image J and K) coursing on the side of the aorta (Image L) ultimately joining the azygos vein (Image M)

**Figure 6:**
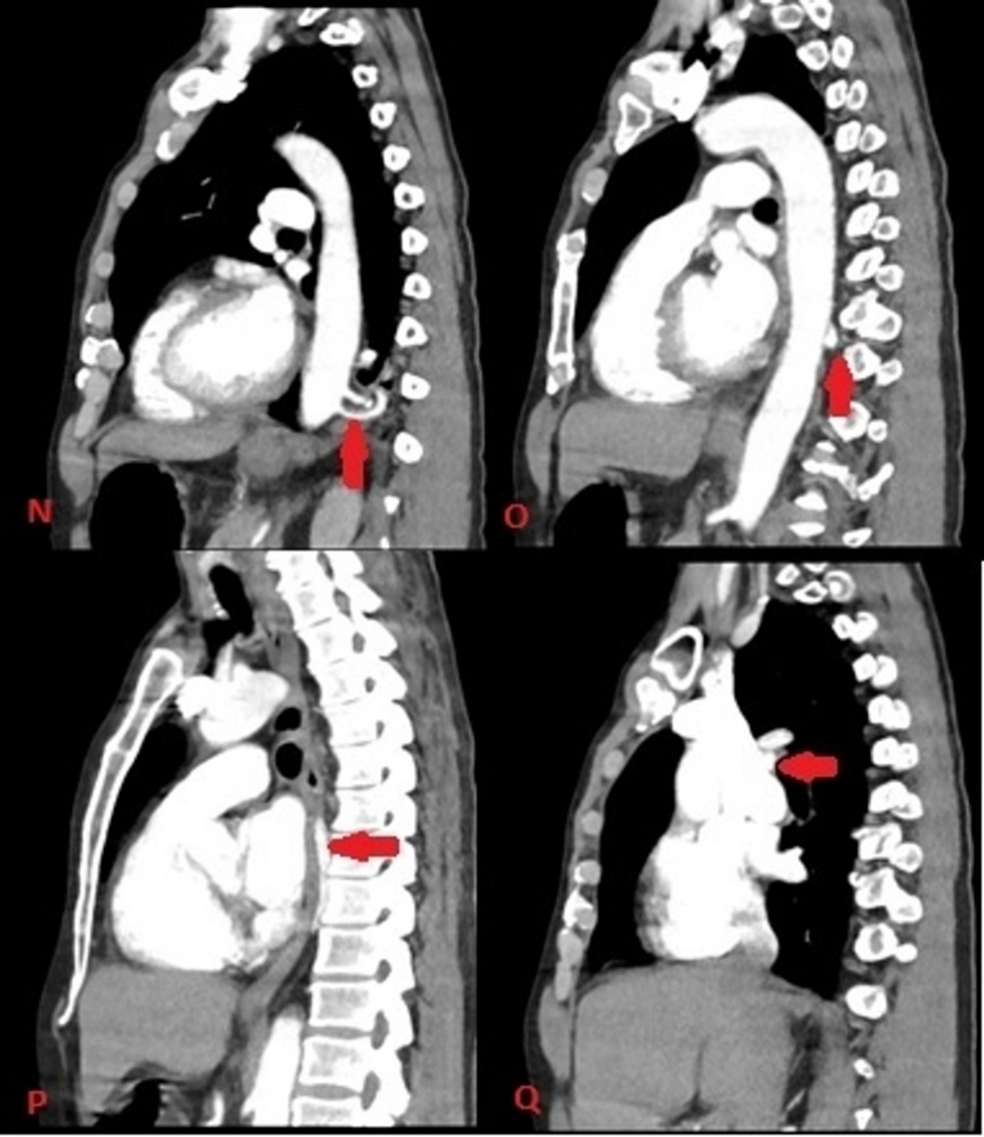
CT of the chest with contrast in the sagittal plane with red arrow showing feeding artery arising from the aorta (Image N), the pulmonary vein coursing around the side of aorta (Image O and P) and ultimately joining the azygos system (Image Q)

A 65-year-old African American man with a past medical history of type 2 diabetes mellitus, chronic obstructive pulmonary disease, and lumbar radiculopathy presented to the emergency department complaining of mild chest pain and right-sided leg swelling with pain for three days. He is an ambulatory, well-nourished, and well-developed man not in acute distress. His vitals are temperature 97.9 F, blood pressure 118/77 mmHg, pulse rate 63/min, respiratory rate 18/min, and oxygen saturation 97% on room air. He is a chronic smoker with a 40-pack-year history and has a family history of venous thromboembolism. Given that the patient has moderate-to-high pre-test probability and elevated D-dimer of 6967 ng/ml, computed tomography (CT) scan of the chest with contrast was performed to rule out pulmonary embolism. The CT scan did not show evidence of pulmonary embolism, however, it revealed a segment of the left lower lobe with chronic interstitial and cystic changes (Figure 1). Moreover, this segment was noticed to have multiple feeding arteries arising from the aorta and partial anomalous pulmonary venous return draining into the azygos vein (Figures 1-6).

**Figure 7:**
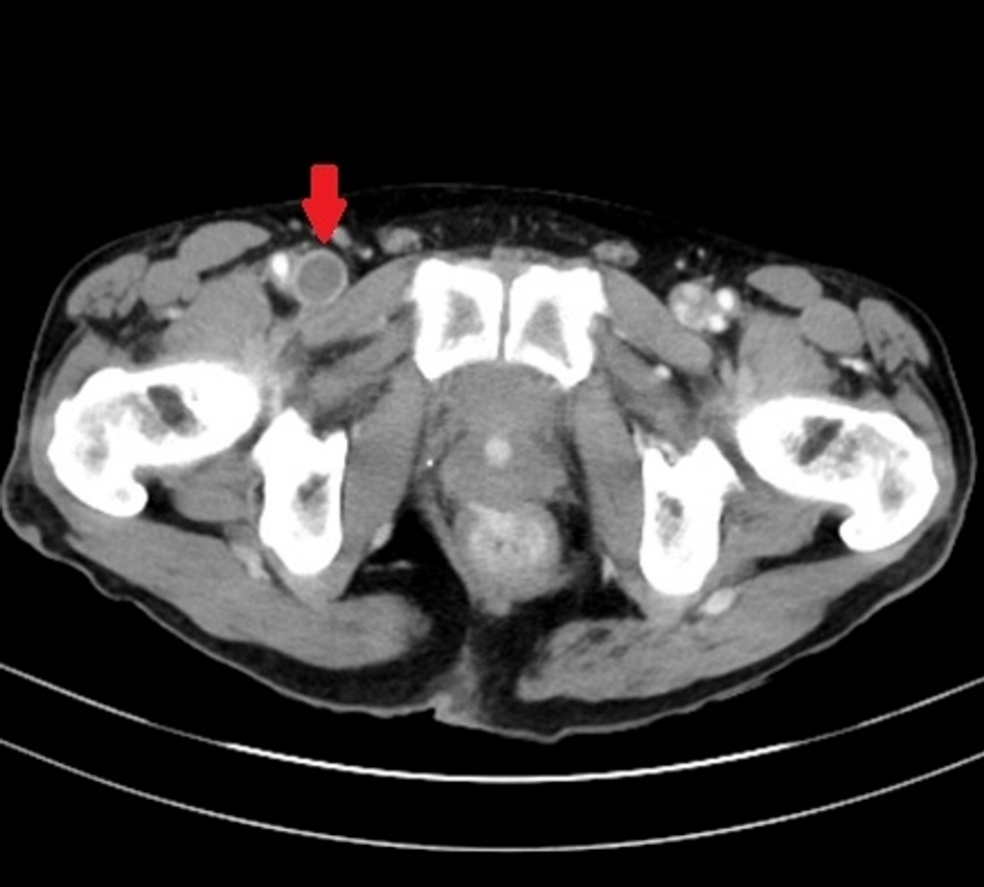
CT of the abdomen and pelvis with contrast in the horizontal plane with the red arrow pointing to deep vein thrombosis of right femoral vein

A diagnosis of intralobar sequestration with anomalous pulmonary venous drainage into the azygos vein was made. The patient reported that he never had any infection in the lungs and was relatively asymptomatic except for chest pain and leg pain that he presented with. As venous thromboembolism was highly suspected, lower extremity Doppler was performed which revealed deep venous thrombosis in the right femoral vein. Incidentally, the clot was also detected on the abdominal CT (Figure 7), which was ordered to better define the anomaly. The patient was started on rivaroxaban for venous thromboembolism. A shared decision was made for continued follow-up in the clinic as he has no pulmonary symptoms and recurrent chest infection.

## Discussion

Bronchopulmonary sequestration is a rare congenital anomaly consisting of non-functioning lung tissue without connection to the tracheobronchial tree [2]. Frequently, it receives blood supply from systemic circulation rather than pulmonary circulation. Based on the pleural encasement, it is classified into intralobar or extralobar sequestration. 

Extralobar sequestration, with its own pleura, is located outside the normal lung. It is usually diagnosed in newborns or prenatally and is associated with other congenital malformations such as congenital diaphragmatic hernia, congenital heart diseases, and vertebral anomalies. Occasionally, it may present as hydrops and is detected on prenatal screening [3]. The initial test of choice in extralobar sequestration is chest radiograph followed by advanced thoracic imaging such as CT and magnetic resonance imaging. Further management whether elective resection or observation depends on risk profiles such as infection, malignancies, pneumothorax, and haemoptysis or haemothorax. 

Intralobar sequestration is located within normal lungs and hence lacks its own pleura. The most common site is usually the left lower lobe although other locations have been described. Although ILS has no functional communication to the tracheobronchial tree, they may have aberrant connections to the lung parenchyma or gastrointestinal tract [4]. These aberrant connections or pores of Kohn act as channels for the entry of bacteria that predisposes them for infection [5]. The arterial supply is usually from systemic circulation and venous drainage is to the left atrium. Our case was an example of intralobar sequestration located in the left lower lobe with feeding arteries from systemic circulation. However, ours was a very rare case where the venous drainage was via anomalous pulmonary vein draining into azygos vein, instead of the left atrium. 

Not uncommonly, intralobar pulmonary sequestration is characterized by recurrent infections, hemoptysis, or pleural effusion, which was not seen in our case. It is often suggested that ILS may have to be resected because of the risk of recurrent infections and considerable shunt through the circuit [5]. 

## Conclusions

We present a rare case of a 65-year-old man presenting with intrapulmonary sequestration with multiple arterial feeds from the aorta and anomalous pulmonary vein draining into the azygous vein. This may be associated with recurrent infections or shunt which may require resection. If asymptomatic, a shared decision may be made with continued surveillance and simple observation. 
